# Relationship between clinicopathological characteristics and CYLD expression in patients with cholesteatoma

**DOI:** 10.1371/journal.pone.0240216

**Published:** 2020-10-08

**Authors:** Shunsuke Miyake, Toru Miwa, Go Yoneda, Ayumi Kanemaru, Haruki Saito, Ryosei Minoda, Yorihisa Orita, Hideyuki Saito, Hirofumi Jono

**Affiliations:** 1 Department of Clinical Pharmaceutical Sciences, Graduate School of Pharmaceutical Sciences, Kumamoto University, Kumamoto, Japan; 2 Department of Pharmacy, Kumamoto University Hospital, Kumamoto, Japan; 3 Department of Otolaryngology-Head and Neck Surgery, Kyoto University, Kyoto, Japan; 4 Department of Otolaryngology-Head and Neck Surgery, Tazuke Kofukai Medical Research Institute Kitano Hospital, Osaka, Japan; 5 Center for Inflammation, Immunity and Infection, Institute for Biomedical Sciences, Georgia State University, Atlanta, Georgia, United States of America; 6 Department of Otolaryngology-Head and Neck Surgery, Kumamoto University Hospital, Kumamoto, Japan; 7 Department of Otolaryngology-Head and Neck Surgery, Kumamoto General Hospital, Kumamoto, Japan; Lund University, SWEDEN

## Abstract

Middle ear cholesteatoma is a destructive disease in which inflammation plays an important role in development and progression, and there are currently no biomarkers predicting prognosis or recurrence. Cylindromatosis (CYLD), a tumor suppressor deubiquitinase, serves as a negative regulator of inflammation expressed in tissues including the middle ear. To determine the clinical significance of CYLD in acquired cholesteatoma, we evaluated CYLD expression in acquired cholesteatoma tissue by immunostaining and analyzed its correlation with clinicopathological characteristics. Our immunohistochemical analysis revealed that CYLD expression levels were varied in the tissues of acquired cholesteatoma patients. The relative expression levels of CYLD in cholesteatoma exhibited a significant correlation with the grade of otorrhea (R = 0.532, *p* = 0.039). Moreover, the period of epithelialization was also significantly associated with the relative expression levels of CYLD (R = 0.720, *p* = 0.002). In addition, CYLD expression tended to be lower in the group with recurrence. These results suggest that low CYLD expression correlates with postoperative recovery of acquired cholesteatoma, while potentially affecting the induction of recurrence. This is the first report showing that low CYLD expression correlates with accelerated disease recovery, and suggests a new aspect of CYLD as a prognostic predictor of acquired cholesteatoma.

## Introduction

Middle ear cholesteatoma is a destructive disease characterized by abnormal growth of keratinized squamous epithelium [1,2]. Chronic inflammation plays an important role in the development and progression of cholesteatoma [3,4]. Prolonged inflammation of the middle ear causes inflammation to the mastoid sinuses and upper tympanic chamber, and poor ventilation of the middle ear causes acquired cholesteatomatous otitis media [5]. When the keratinized tympanic membrane is depressed and reaches deep in the ear, bone destruction, otorrhea, hearing loss, vertigo/dizziness, tinnitus, and facial paralysis are caused. The primary treatment for acquired cholesteatoma is surgery [6], and the detailed treatment methods are determined according to the patient's condition such as the degree of acquired cholesteatoma. There are various methods for classifying the degree of progression of acquired cholesteatoma, such as those proposed by Meyerhoff [7], Saleh & Mills [8], and Japan Otological Society (JOS) [9]. However, there is still no widely accepted classification for the acquired cholesteatoma in the world, and there are no biomarkers available for treatment selection and prognosis as used for other diseases. To date, altered expression of several tumor suppressor genes (e.g., p53, p27) has been reported in acquired cholesteatoma, and the link to pathogenesis has been investigated [10,11]. Nevertheless, no clear correlation between the gene expression and prognosis has been obtained in acquired cholesteatoma. To improve the treatment of acquired cholesteatoma, clinically-useful biomarkers are urgently required.

Cylindromatosis gene (*CYLD*), a tumor suppressor gene, was initially identified as a mutated gene involved in familial cylindromatosis, a skin tumor that develop from skin appendages [12]. CYLD protein has a ubiquitin-specific (USP) domain in the C-terminal region and functions as a deubiquitinase [13]. CYLD has been reported to suppress various signals such as NF-κB, and has a function as a negative regulator of inflammation [14–16]. In clinical practice, it has been reported that loss of CYLD expression correlates with progression and prognosis in several types of cancer [17–25]. Recently, CYLD is expected to be useful as a marker for prognosis prediction. In addition to its usefulness as prognosis biomarkers in various cancers, it has been reported that CYLD is also expressed in the middle ear and plays important roles in regulating inflammatory response [26]. Indeed, the loss of CYLD expression in the middle ear caused the excess inflammatory response, the typical hallmark of acquired cholesteatoma [26], suggesting the possibility that CYLD could be a marker of progression and prognosis in inflammatory disease acquired cholesteatoma.

In this study, we evaluated CYLD expression in cholesteatoma tissue by immunostaining and assessed the clinical data of patients to determine the clinical significance of CYLD in acquired cholesteatoma. Furthermore, we analyzed the relationship between CYLD expression level and clinicopathological characteristic in cholesteatoma.

## Materials and methods

### Patients

A series of 16 acquired cholesteatoma tissues were obtained during cholesteatoma surgery from January 2016 to January 2019. At surgery, the cholesteatoma was completely removed by surgeons. The paired 16 retroauricular (RA) skin tissues were used as control. This study was approved by the Institutional Review Board of Kumamoto University Hospital (approved number: No.14062). Written informed consent was obtained from all participants in this investigation. In case the patient is a minor, the informed consent was obtained from parents or guardians.

### Immunohistochemistry

The cholesteatoma and RA skin tissues were fixed in 4% paraformaldehyde. Tissues were snap frozen and embedded in OCT compound (Sakura Finetek, Torrance, CA, USA). Tissues were sectioned at a thickness of 10 μm and mounted on slides (Matsunami, Osaka, Japan). Tissue sections were digested with proteinase K (Agilent, Santa Clara, CA, USA) for 15 minutes at room temperature, followed by being washed with phosphate-buffered saline (PBS). Endogenous peroxidase activity was blocked with 3% hydrogen peroxide in methanol for 15 minutes, followed by being washed with PBS. Subsequently, tissue sections were incubated with non-specific staining blocking reagent (Nacalai tesque, Kyoto, Japan) for 20 minutes at room temperature, and incubated with 200x diluted primary rabbit anti-CYLD polyclonal antibody (Sigma, St. Louis, MO, USA) overnight at 4°C. Then, tissue sections were incubated with secondary antibodies for 60 minutes at room temperature, followed by being washed with PBS. For visualization, DAB Substrate Kit (Agilent) was used according to the manufacturer’s instructions, followed by counterstaining with Mayer’s hematoxylin (Wako, Osaka, Japan).

### Analysis of immunohistochemical image

Using an Olympus (Tokyo, Japan) light microscope, 5 representative areas of each slide were photographed (magnification, x400). The percentage of the area where CYLD was positive in the epithelium was determined using WinRoof (Mitani, Fukui, Japan), and the average value of 5 areas was calculated. The values obtained are defined as “CYLD%”. CYLD% in cholesteatoma was corrected by that in RA skin to obtain the relative expression level of CYLD, which was used for analysis.

### Clinical analysis

The stage of acquired cholesteatoma was preoperatively determined according to the JOS staging system for middle ear cholesteatoma [9]. After the surgery, patients were followed up for one year. Otorrhea was confirmed by otoendoscopy and classified into a grade of 0 to 3 based on observation by the surgeon before 1 day and after surgery 1 year. The period of epithelialization was measured as the number of days since cholesteatoma surgery required for the tympanic membrane to become epithelialized completely. The recurrence was evaluated on 1 year after surgery, which was based on whether or not the recurrence of cholesteatoma was diagnosed by otoendoscopy and CT scan.

### Statistical analysis

All statistical analyses were performed using Statcel-the Useful Addin Forms on Excel-2nd ed. software (OMS Publishing, Tokyo, Japan). The Wilcoxon signed rank test was used to compare the means of paired variables, and the Mann-Whitney U test was used to compare the means of unpaired variables. The Spearman’s rank order correlation was used to assess the correlation between the relative expression level of CYLD and the grade of otorrhea. The correlation of the relative expression level of CYLD with the period of epithelialization was analyzed with the Pearson’s correlation test. The *p* value of <0.05 was considered to be significant.

## Results

### CYLD expression in RA skin and cholesteatoma tissues

**Fig 1:**
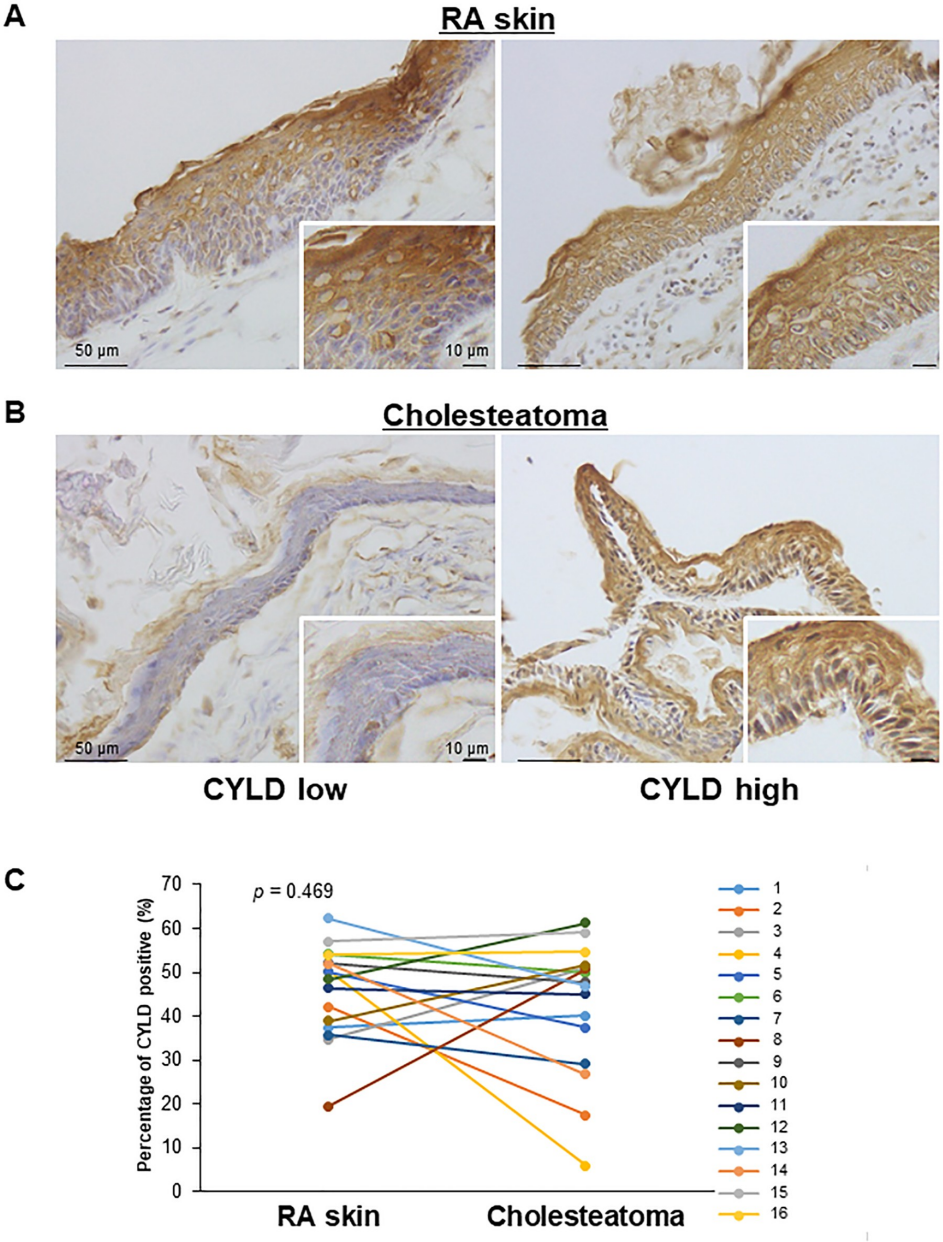
Immunohistochemical analysis of CYLD expression in cholesteatoma. Representative CYLD immunohistochemical images in RA skin as control tissues (A) and cholesteatoma (B) in the patient with low CYLD expression (left panel) or high CYLD expression (right panel). Original magnification: x400. (C) The percentage of area showing CYLD positive in the RA skin and cholesteatoma epithelium. Each line shows CYLD expression in RA skin and cholesteatoma epithelium by individual patients. The numbers shown to the right of the graph indicate the patient numbers.

To determine the clinical significance of CYLD expression in cholesteatoma, we first performed immunohistochemical analysis of CYLD in the tissues obtained from 16 patients with acquired cholesteatoma. As shown in Fig 1A, CYLD expression was mainly detected in the cytoplasm of the spinous and granular layer in almost all RA skin tissues, as control tissues. In contrast, in cholesteatoma, although CYLD expression was also observed in the cytoplasm of the spinous and granular layers, the expression levels were varied in the tissues of cholesteatoma patients (Fig 1B). Intriguingly, some patients exhibited low CYLD expression in cholesteatoma (Fig 1B left panel: CYLD low) and others showed high expression (Fig 1B right panel: CYLD high). The percentage of area showing CYLD positive in RA skin epithelium was 19–62%, while that in cholesteatoma epithelium was 5–61% (Fig 1C). There was no significant difference between the percentage of the area where CYLD was positive in RA skin epithelium and that in cholesteatoma epithelium (*p* = 0.469).

### Clinical significance of CYLD expression in cholesteatoma

**Fig 2:**
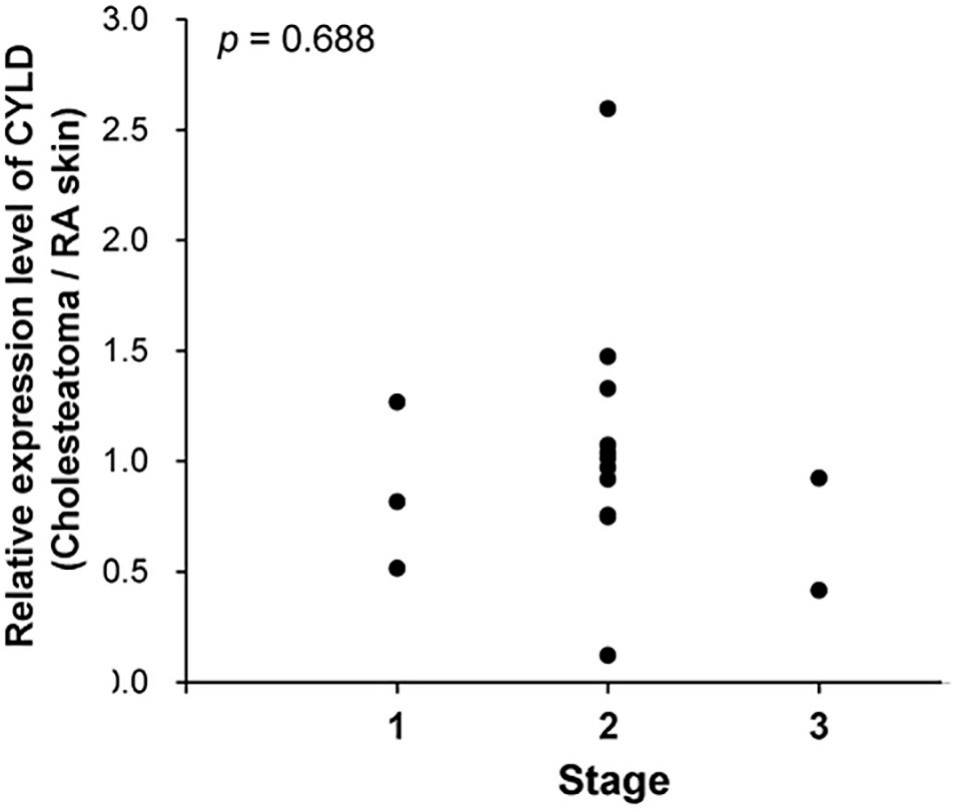
The relationship between the relative expression level of CYLD in cholesteatoma and the stage. Plots showing the relationship between CYLD expression in cholesteatoma and the stage. The stage was decided by JOS staging system.

Next, we evaluated the clinical data of patients to determine the pathological significance of CYLD expression in cholesteatoma. The mean age of cholesteatoma patients was 47.8 (15–77 years, Table 1). There were 11 males and 5 females. All patients had no otorrhea and inflammation before the surgery. The relative expression levels of CYLD in cholesteatoma did not significantly correlate with the stage of cholesteatoma (*p* = 0.688, Fig 2), suggesting that CYLD expression was not associated with the progression of cholesteatoma.

**Table 1:** Clinical data of 16 patients with acquired cholesteatoma.

No.	Age	Sex	Stage	Period of epithelialization (days)	Otorrhea (Class)	Recurrence	CYLD% (RA skin)	CYLD% (Cholesteatoma)
1	71	F	2	15	2	+	37.47	40.17
2	77	M	3	22	0	-	42.08	17.40
3	33	M	2	125	3	-	34.63	51.00
4	59	F	2	22	1	+	50.22	5.97
5	76	M	2	56	1	-	50.14	37.45
6	72	M	3	74	2	-	54.17	49.93
7	17	M	1	25	0	-	35.66	29.08
8	32	F	2	197	2	-	19.53	50.69
9	15	M	2	6	0	-	52.07	47.70
10	56	M	2	59	0	-	38.96	51.68
11	26	M	2	18	0	-	46.33	44.94
12	30	F	1	206	3	-	48.39	61.28
13	38	F	2	59	0	+	62.26	46.93
14	75	M	1	7	0	+	52.03	26.69
15	54	M	2	7	0	-	57.07	59.10
16	34	M	2	55	2	-	54.00	54.63

“No.” corresponds to the patient number in Fig 1C. Recurrence: (+) means there was recurrence, (-) means no recurrence.

### Correlations between CYLD expression and the clinicopathological characteristic in cholesteatoma patients

**Fig 3:**
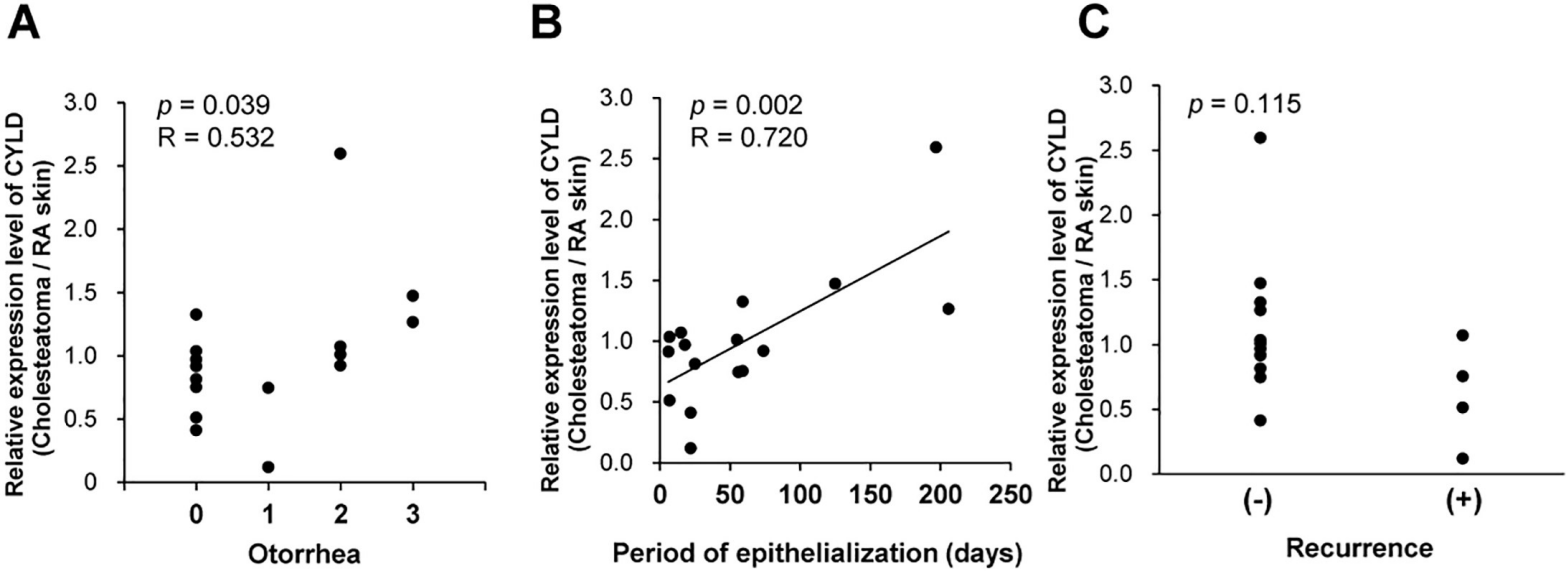
Correlations between the relative expression level of CYLD and the clinicopathological characteristic in cholesteatoma. (A-C) Correlations between the relative expression level of CYLD in cholesteatoma with the grade of otorrhea (A), the period of epithelialization (B), and the recurrence (C).

Based on the results shown in Figs 1 and 2 and Table 1, we further performed a correlation analysis to determine whether the relative expression levels of CYLD correlated with clinicopathological characteristic in cholesteatoma patients. As shown in Fig 3A, the relative expression levels of CYLD in cholesteatoma exhibited a significant correlation with the grade of otorrhea (R = 0.532, *p* = 0.039). Moreover, the period of epithelialization was also significantly associated with the relative expression levels of CYLD (R = 0.720, *p* = 0.002, Fig 3B). These results suggest that low CYLD expression correlates with mild symptoms and rapid postoperative recovery. In case we focused on the correlation with the presence or absence of recurrence, the relative expression level of CYLD in cholesteatoma tended to be lower in the group with recurrence, although it was not significant (*p* = 0.115, Fig 3C). It should be noted that, the stage of cholesteatoma did not significantly correlate with any of the grade of otorrhea (*p* = 0.320), the period of epithelialization (*p* = 0.450) or the recurrence (p = 0.303). These results suggest the possibility that the relative expression levels of CYLD in cholesteatoma tissues may be a novel molecular marker for indicating the clinicopathological characteristic in cholesteatoma patients.

## Discussion

In this study, we revealed the possibility that CYLD expression in acquired cholesteatoma correlates with clinicopathological characteristics, especially progression preoperatively and prognosis including infection, wound healing and recurrence in cholesteatoma patients.

One of the novelties of this study is that, CYLD expression in acquired cholesteatoma tissue significantly correlated with the clinicopathological characteristic in patients with cholesteatoma. As shown in Fig 3, the relative expression levels of CYLD in cholesteatoma exhibited a significant correlation with the grade of otorrhea (R = 0.532, *p* = 0.039, Fig 3A) and the period of epithelialization (R = 0.720, *p* = 0.002, Fig 3B). Low otorrhea grade indicates milder symptoms and shorter period of epithelialization indicate faster postoperative healing. These results suggest that CYLD expression may be a molecular marker for the grade of acquired cholesteatoma symptoms and its subsequent recovery. For patients who are expected to slower recovery due to high CYLD expression, additional treatment may be considered. It is necessary to investigate the mechanism by which CYLD expression in acquired cholesteatoma epithelium affects the prognosis after surgical removal of acquired cholesteatoma tissue. The mechanism was speculated as following; cytokines which were released from the cholesteatoma epithelium [27–29] caused strong inflammation, resulting in high CYLD expression to suppress the inflammation. However, removal of cholesteatoma by surgery influenced on CYLD expression in surrounding tissues. In case inflammation around the cholesteatoma after removal was remained, CYLD expresses high to suppress inflammation. While, in case inflammation around the cholesteatoma after removal was not remained, low CYLD expression decreased keratinocyte differentiation, and increased proliferation (i.e. wound healing) [30]. Although it is still unclear whether NF-κB signaling is involved in the “recovery” of acquired cholesteatoma postoperatively, in brief, we speculated that high CYLD worked as suppressor of inflammation and low CYLD worked as initiator of wound healing. In addition, the relative expression level of CYLD in cholesteatoma tended to be lower in the group with recurrence, although it is not significant (*p* = 0.115, Fig 3C). This indicated that CYLD expression in acquired cholesteatoma tissues may be an independent factor for recurrence in acquired cholesteatoma. In general, acquired cholesteatoma is likely to recur when there is a residue, and it has been reported that more advanced acquired cholesteatomas are more likely to recur [6]. However, some cases without residue recur at an early stage postoperatively, and the indicators for predicting recurrence are not sufficient. To the best of our knowledge, there are no molecular markers that predict recurrence of acquired cholesteatoma. Previous reports indicated that reduced expression of CYLD in acquired cholesteatoma causes NF-κB activation [31], which contributes to excessive proliferation of keratinocytes [32]. These reports suggested low CYLD was related to the progression of cholesteatoma. However, our results suggested that low CYLD affected recurrence, not progression. These might be caused by as following reasons; the stage of progression was the results of excessive proliferation of keratinocytes, but it did not reflect the real time activation of proliferation. In brief, the timings of CYLD evaluation was the same, but the evaluation items were different each other (i.e. stage of progression: “results” or proliferation: “cause”). The mechanism by which CYLD expression is associated with recurrence is uncertain. Proliferation markers should be considered in future studies to determine why recurrence occurs. Unlike the results in the previous section, there was no significant difference in recurrence, so further careful examination is required. However, CYLD expression may be useful as a new marker for predicting recurrence.

Another novelty of this study is that, contrary to our expectations, low CYLD expression in acquired cholesteatoma correlated with improved grade of otorrhea and shorter days to epithelialization. In recent studies, low CYLD expression has been reported in acquired cholesteatoma tissue [31] and in several carcinomas, such as breast [23], oral [25], and liver [22]. In addition, it has been shown that low CYLD expression correlates with the progression of disease and poor prognosis via activation of NF-κB [23] and TGF-β signals [25]. In contrast, the present study showed that low CYLD expression may alleviate symptoms and promote postoperative recovery in acquired cholesteatoma. As of this moment, this is the first report to suggest the possibility that low CYLD expression may correlate with disease recovery. It is well-documented that the chronic inflammation plays an important role in the development and progression of cholesteatoma [3,4]. Prolonged inflammation of the middle ear causes inflammation to the mastoid sinuses and upper tympanic chamber, and poor ventilation of the middle ear causes acquired cholesteatomatous otitis media. Because it has been reported that CYLD expression is markedly up-regulated by inflammatory insults in middle ear [26], it is possible that CYLD expression was up-regulated in acquired cholesteatoma patients with strong inflammation postoperatively, resulting in otorrhea and slow recovery. Furthermore, as mentioned above, low CYLD expression may promote cell proliferation and accelerate recovery. Thus, these results suggest a new aspect of CYLD, in which low CYLD expression may be involved in disease recovery as well as poor prognosis in cancer. Further studies will focus on elucidating the detailed roles of CYLD expression in the pathogenesis of cholesteatoma. Taken together, we speculated that high/low CYLD expression regulates inflammation and keratinocyte differentiation/proliferation in the various stage from infection-wound healing-tumorigenesis in the middle ear.

Our present study has several drawbacks. Firstly, CYLD value was not examined in the middle ear mucosa pre- and postoperatively. Secondarily, Follow-up term was only one year after surgery. Since this is a preliminary study with a limited number of cases, further study is needed to verify the usefulness of CYLD expression as a novel molecular marker for indicating the clinicopathological characteristic in cholesteatoma patients, and elucidate the roles of CYLD expression in the pathogenesis of cholesteatoma

In conclusion, the present study revealed that low CYLD expression in acquired cholesteatoma may correlate with improvement of symptoms and early recovery, while causing recurrence. Further investigation focusing on CYLD expression may contribute to elucidating the detailed molecular pathogenesis of cholesteatoma and open up new insights into improving the treatment of acquired cholesteatoma.
